# Toluidine blue staining as an adjunctive tool for early diagnosis of dysplastic changes in the oral mucosa

**DOI:** 10.4317/jced.51121

**Published:** 2013-10-01

**Authors:** Shambulingappa Pallagatti, Soheyl Sheikh, Amit Aggarwal, Deepak Gupta, Ravinder Singh, Roopika Handa, Simranpreet Kaur, Jyoti Mago

**Affiliations:** 1 M.D.S., Professor, Department of oral medicine and radiology, M.M. College of Dental Sciences and Research, Mullana, Ambala, Haryana, India; 2 M.D.S., Assistant Professor, Department of oral medicine and radiology, M.M. College of Dental Sciences and Research, Mullana, Ambala, Haryana, India; 3 Post Graduate Student, Department of oral medicine and radiology, M.M. College of Dental Sciences and Research, Mullana, Ambala, Haryana, India

## Abstract

Prognosis of oropharyngeal squamous cell carcinoma depends on early diagnosis, despite advanced surgical techniques, the 5-year survival rate remains ~40-50%. Unfortunately, it is usually detected when it becomes symptomatic. This requires treatment which gives rise to a high rate of morbidity and mortality and, furthermore, early detection of oro-pharyngeal pre-malignant lesions is important to improve the survival rate and quality of life. Since dysplasia and in situ carcinoma contain much more DNA and RNA than the normal surrounding epithelium, the use of in vivo staining, by means of toluidine blue dye, is based on the fact that it is an acidophilic dye that selectively stains acidic tissue components such as DNA and RNA. Toluidine blue staining is considered to be sensitive in identifying early oro-pharyngeal premalignant and malignant lesions. In the present study, the use of toluidine blue staining was taken into consideration to identify clinically doubtful oro-pharyngeal lesions and to compare toluidine blue stain and with the histological evaluation.

** Key words:**Early detection, improved survival, pre-cancer, toluidine blue, vital staining.

## Introduction

Malignancies of the upper aero digestive tract are one of the common malignancies in the world (1). In India, for example, oral cancers constitute 40% of all cancers and rank as the most common cancer in men and third most common cancer in women (2,3). Tumors arising from the oral and oropharyngeal malignancies are usually well advanced at the time of diagnosis (1). Prognosis of oropharyngeal squamous cell carcinoma (oral cavity and pharynx) depends on early diagnosis, despite advanced surgical techniques and adjuvant treatment, the 5-year survival rate remains ~ 40-50% (4). The disease is life threatening, with high morbidity resulting from late treatment. However, if it is diagnosed at an early stage, oral cancer is often curable and inexpensive to treat (1).

The clinical examination is one of the best modalities in suspecting the pathology but the biggest disadvantage in the diagnosis lies in deciding the site of biopsy in early lesions and sometimes whether or not a biopsy is required in these lesions. Early stages are difficult to detect as the lesion may not be palpable and colour changes are not very different from the colour of the surrounding mucosa. Thus identifying clinically suspicious/undetectable lesions has gained importance whereby diagnosis can be confirmed by biopsy at an earlier stage (1).

In developing countries such as India, where there is high prevalence of disease, the focus is on down staging oral cancer at diagnosis from advanced to earlier disease. It is assumed that if premalignant lesion is detected and treated, the lesion may not progress to cancer (5).

In the past decades, adjunctive techniques have emerged with claims of enhancing the oral mucosal examinations and facilitating the detection of and distinctions between oral benign and oral premalignant and malignant lesions (5).

Techniques that are promoted or assessed to improve earlier detection and diagnosis of oral malignancy include toluidine blue, vizilite plus with touidine blue, velscope brush biopsy, etc. The gold standard diagnostic test for oral mucosal lesions that are suggestive of pre malignancy or malignancy remains tissue biopsy and histopathological examination.

Toluidine blue is a cationic metachromatic dye that may selectively bind to free anionic groups such as sulfate, phosphate, and carboxylate radicals of large molecules (6). It has been used for decades as an aid to the identification of mucosal abnormalities of the cervix as well as in the oral cavity. It has been valued by surgeons as a useful way of demarcating the extent of a lesion prior to excision (7).

When evaluated as a part of the clinical examination, toluidine blue staining may provide additional information. In vivo, toluidine blue stains deoxyribonucleic acid and/or may be retained in intracellular spaces of dysplastic epithelium and clinically appear as royal blue areas (6). Toluidine blue staining is considered to be sensitive in identifying early oral premalignant and malignant lesions (4). Therefore, the aim of this study was to identify dysplastic changes undergoing in oral mucosal lesions with toluidine blue staining and to compare the results of toluidine blue stain with histopathological evaluation.

## Material and Methods

The study was focused on 40 oral mucosal lesions in 32 patients, who reported to the Department of Oral Medicine and Radiology, Maharishi Markandeshwar College of Dental Sciences and research, Mullana. Ethical approval was taken from the university ethical committee according to Declaration of Helsinki. Patients who clinically presented with at least one oral mucosal lesion suspected of dysplastic changes and the patients who gave their consent for the study were included in the study. During history taking, information regarding habit of tobacco, its type, duration and frequency was recorded by asking the patient.

Patient who had presence of frank malignancy or those who were suffering from any systemic disease that interfere with or are contraindications to biopsy procedure or undergoing any dental treatment such as orthodontic appliances or prosthesis that may interfere with the examination, were excluded from the study.

All the lesions were first identified under visual examination. This was followed by an oral rinse with 1% acetic acid solution which was given to the patient to hold in the mouth for 20 seconds before expectorating. Toluidine blue (1% W/W) was applied as an oral rinse for 20 seconds and then 1% acetic acid was used for 20 seconds to eliminate mechanically retained stain. Only the dark staining lesions were taken as positive whereas equivocal stains (faint stains) and the lesions which did not retain the stain at all were taken as negative but all the lesions were subjected to histopathological examination.

The biopsies were performed under local anaesthesia. The pathologists examining all the biopsies were not informed regarding the clinical or staining evaluation of each sample. Histopathologic diagnoses were referred as: non-neoplastic (hyper-keratoses, hyper-para-keratoses, etc), mild dysplasia, moderate dysplasia, severe dysplasia, squamous cell carcinoma. For the statistical analysis, we used histopathologic assessment as the gold standard to which toluidine blue stains were compared.

## Results

**Figure 1:**
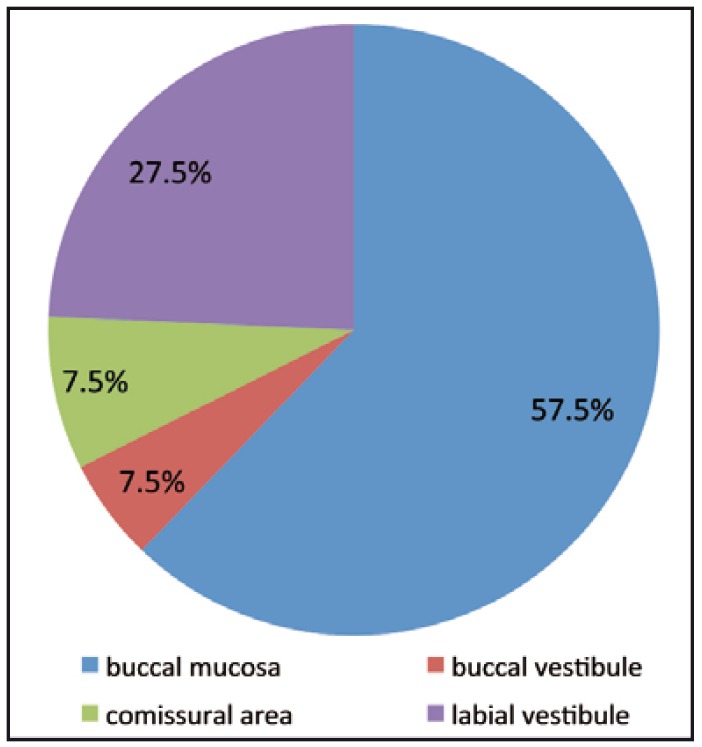
Common site of occurrence of precancerous lesions.

Out of 32 patients examined, 29 were males and 3 were females. Most common site where the lesion was present was buccal mucosa in 57.5% of the total cases (23 lesions) followed by labial vestibule 27.5% (11 lesions) and commissural area and buccal vestibule 7.5% each (3 lesions) (Fig. 1).

**Figure 2:**
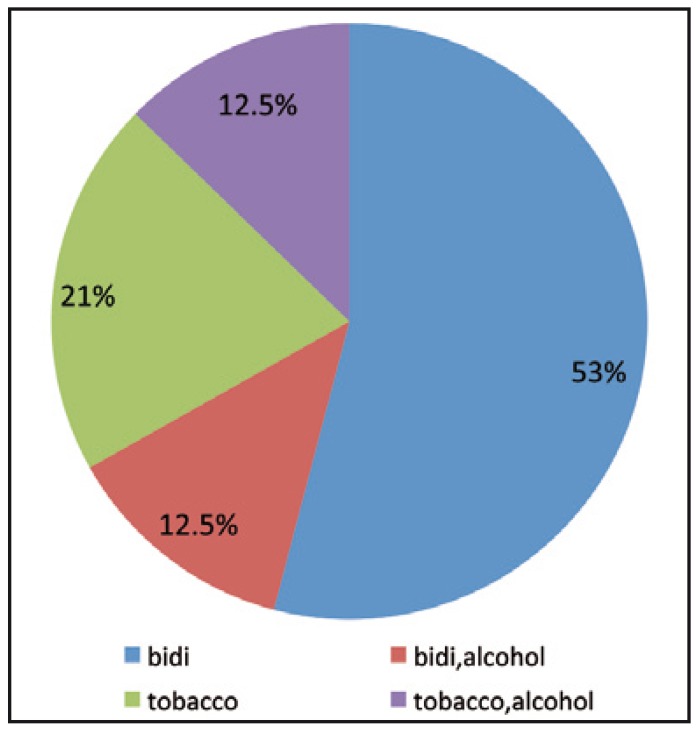
Distribution of habit amongst patients.

Further it was observed that the most common habit amongst them was smoking tobacco which was seen in 17 patients (53%) followed by smokeless tobacco in 7 patients (21%) and smoking and smokeless tobacco along with alcohol in 4 patients (13%) (Fig. 2).

**Figure 3:**
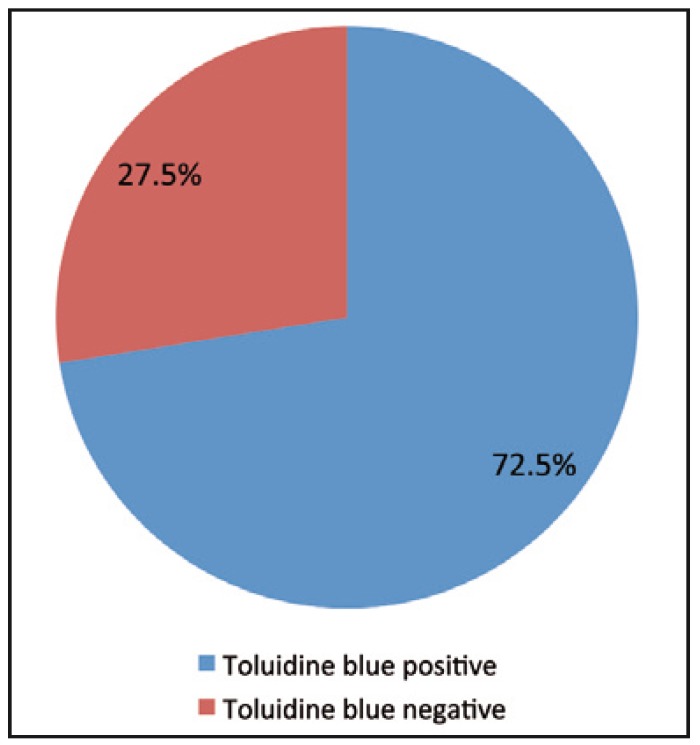
Results of toluidine blue staining.

The results of toluidine blue staining showed that 29 (72.5%) out of the total 40 oral mucosal lesions stained positive while 11 (27.5) were negative (Fig. 3).

Histopathological examination of the 40 oral mucosal lesions showed 3 non-conclusive reports. Out of the 37 conclusive reports, 14 (37.83%) were benign lesions (hyperkeratosis, hyperparakeratosis, epithelial and basilar hyperplasia) and 23 were precancerous or cancerous lesions. Out of the 23 precancerous lesions, 13 (56.5%) were mild dysplasia and 10 (43.47%) were moderate dysplasia ( Table 1).

**Table 1:**
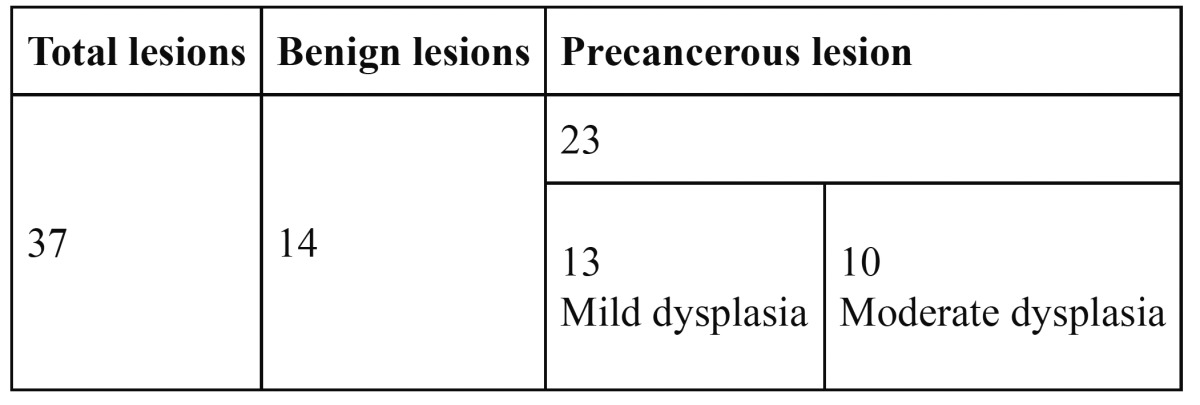
Histopathological examination of conclusive reports.

The results of the toluidine blue staining and histological findings are outlined in  Table 2. When toluidine blue staining and histopathology were compared, it was observed that 10 (90.9%) out of the total 11 toluidine blue negative lesions were histologically benign lesions while 22 (84.6%) out of the 26 toluidine blue positive lesions were histologically defined as pre-cancerous or cancerous lesions.

**Table 2:**
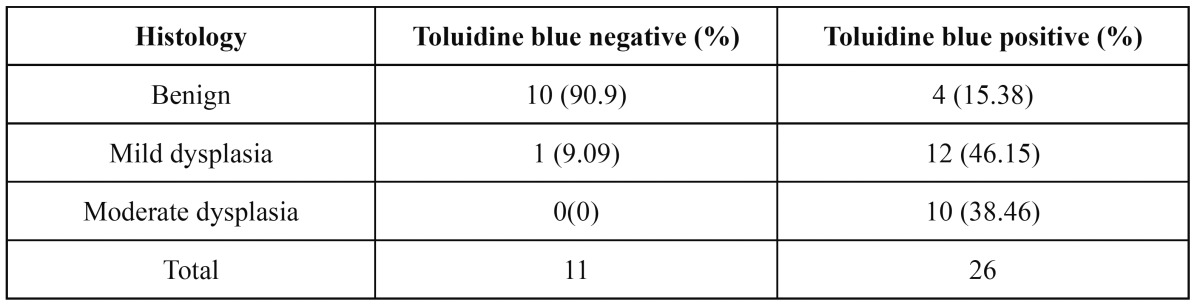
Correlation between toluidine blue staining & histopathology.

The results of the toluidine blue test and histology, were compared in order to calculate the sensitivity (true-positivity) and specificity (true-negatives) ( Table 3). The sensitivity for toluidine blue staining was 95% and specificity was 71.45%. The positive predictive value for toluidine blue staining was 84.6% and the negative predictive value was 90.9% and the diagnostic accuracy was 86.48%.

**Table 3:**
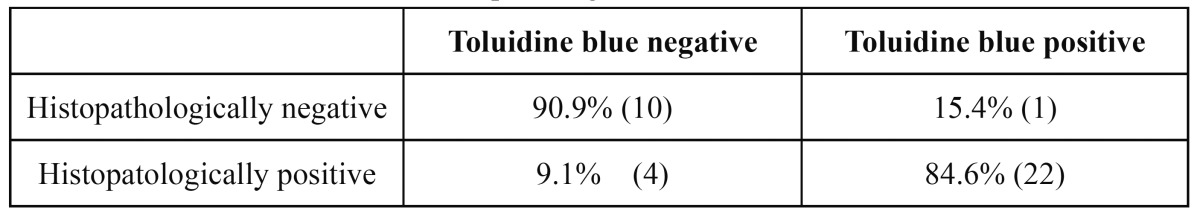
Toluidine blue stain and histopathological correlation.

## Discussion

The toluidine blue literature shows that it is a practical, rapid, inexpensive, and effective adjunct diagnostic tool (8). Toluidine blue, also known by its chemical name tolunium chloride, is a basic metachromatic dye that is known for its property of differentially staining malignant neoplasm but not normal epithelium. It is postulated that the increased amount of DNA and RNA in neoplastic cells and the wider intercellular canals compared to normal epithelial cells are responsible for staining malignant cells (9). Its clinical application in staining neoplastic cells was first described by Richart in 1963 (10) who used the dye to stain cervical carcinoma in situ. Since then it has emerged as a vital stain for detection of cervical dysplasia and carcinoma during colposcopy (9). More recently it has been recommended as an adjunctive method to assist in early detection of oral premalignant lesions and oral squamous cell carcinoma, to assist biopsy site selection, for assessment of margins of oral premalignant lesions/ oral squamous cell carcinoma and in determining oral premalignant lesion at risk of progression to carcinoma (8).

In all fairness its ability to detect malignant oral cavity lesions has been well documented in clinical settings (8,9). Studies assessing Toluidine blue have shown a sensitivity and specificity ranging from 93.5 to 97.8% and 73.3 to 92.9% respectively (11).

Epstein et al. (12) while screening for recurrence in patients who had previously been treated for upper aero-digestive tract malignancies, found that the use of tolonium chloride rinse is more sensitive than clinical examination alone in detecting lesions that might be found on biopsy to be carcinoma or carcinoma in situ. They reported that the sensitivity increased with its use in detecting neoplasm as compared to unaided clinical exam from 26.6% to 96.7%. The increased sensitivity is largely attributed to lesions that stain but were not detected clinically on visual examination (12). These findings were similar to those observed in our study where we found out sensitivity and specificity of toluidine blue staining in detecting premalignant/ malignant lesions was 95% and 71.4% respectively and the diagnostic accuracy of toluidine blue when compared with histopathological evaluation came out to be 86.48%. Similar findings were observed by Allegra et al. (4) who conducted a similar study on toluidine blue and reported a sensitivity of 96.2% and a specificity of 77.7%.

The negative and the positive predictive value came out to be 90.9% and 84.6% respectively which was in accordance with the study conducted by Allegra et al. (4). Therefore, it was observed that toluidine blue might provide utility in reducing the number of biopsies by approximately half while identifying all lesions represen-ting severe dysplasia and Oral Squamous Cell Carcinoma.

The predictive values of a positive test for clinical examination and tolonium chloride staining were similar (36.4% vs 32.6%; p = .5871), indicating that the greater sensitivity of tolonium chloride was not associated with an excessive number of unnecessary biopsies (false positives) (12). Therefore, toluidine blue negative lesions need not to be subjected to further histopathological examination, thus saving time and resources.

Furthermore, it was observed that while comparing the dysplatic changes occurring at various sites with the histopathology, no statistically significant differences were observed (p=0.277), which depicted that it could not be used as a tool for site specific dysplastic changes.

Toluidine blue is useful in raising or confirming clinical suspicion of malignancy or pre-malignancy and has the capability to reduce the number of biopsies being done. It has been proved in our study that when the lesion stained faintly, it came out to be histopathologically negative in most cases. Though our findings suggest that toluidine blue staining may be considered as an adjunctive diagnostic tool for detecting dysplastic changes in the epithelium but larger studies are required to assertively and definitively answer the questions related to the screening use of toludine blue stains as early detection tool.
